# Validation of coding algorithms for the identification of patients hospitalized for alcoholic hepatitis using administrative data

**DOI:** 10.1186/s12876-015-0348-5

**Published:** 2015-09-11

**Authors:** Jack XQ Pang, Erin Ross, Meredith A. Borman, Scott Zimmer, Gilaad G. Kaplan, Steven J. Heitman, Mark G. Swain, Kelly W. Burak, Hude Quan, Robert P. Myers

**Affiliations:** 1 Liver Unit, Division of Gastroenterology and Hepatology, University of Calgary, Calgary, AB Canada; 2 Department of Community Health Sciences, University of Calgary, Calgary, AB Canada; 3 Medical Services, Alberta Health Services, Calgary, AB Canada

## Abstract

**Background:**

Epidemiologic studies of alcoholic hepatitis (AH) have been hindered by the lack of a validated International Classification of Disease (ICD) coding algorithm for use with administrative data. Our objective was to validate coding algorithms for AH using a hospitalization database.

**Methods:**

The Hospital Discharge Abstract Database (DAD) was used to identify consecutive adults (≥18 years) hospitalized in the Calgary region with a diagnosis code for AH (ICD-10, K70.1) between 01/2008 and 08/2012. Medical records were reviewed to confirm the diagnosis of AH, defined as a history of heavy alcohol consumption, elevated AST and/or ALT (<300 U/L), serum bilirubin >34 μmol/L, and elevated INR. Subgroup analyses were performed according to the diagnosis field in which the code was recorded (primary vs. secondary) and AH severity. Algorithms that incorporated ICD-10 codes for cirrhosis and its complications were also examined.

**Results:**

Of 228 potential AH cases, 122 patients had confirmed AH, corresponding to a positive predictive value (PPV) of 54 % (95 % CI 47–60 %). PPV improved when AH was the primary versus a secondary diagnosis (67 % vs. 21 %; *P* < 0.001). Algorithms that included diagnosis codes for ascites (PPV 75 %; 95 % CI 63–86 %), cirrhosis (PPV 60 %; 47–73 %), and gastrointestinal hemorrhage (PPV 62 %; 51–73 %) had improved performance, however, the prevalence of these diagnoses in confirmed AH cases was low (29–39 %).

**Conclusions:**

In conclusion the low PPV of the diagnosis code for AH suggests that caution is necessary if this hospitalization database is used in large-scale epidemiologic studies of this condition.

## Background

Alcoholic hepatitis (AH) is a clinical syndrome characterized by hepatic dysfunction in the setting of heavy alcohol intake. Rapid onset of jaundice is a cardinal manifestation of AH; other common signs include fever, ascites, muscle wasting and hepatic encephalopathy [1]. AH is often complicated by infection and hepatorenal syndrome, both of which significantly increase mortality [2–4]. Untreated patients with severe AH, typically defined by a Maddrey discriminant function (DF) ≥32 and/or the presence of hepatic encephalopathy, have a particularly poor prognosis with one-month mortality rates ranging from 30 % to 50 % [5, 6]. In patients with a mild presentation of AH, the risk of progression to cirrhosis is 50 %; this risk is highest in patients who continue to abuse alcohol [7].

Population-based studies describing the epidemiology and outcomes of AH are limited. A Danish study by Sandahl et al. [8] was the first population-based epidemiologic study of AH and demonstrated an increase in annual incidence among both Danish men and women between 1999 and 2008. Another study described the clinical characteristics and mortality of patients hospitalized in the United States for AH using the Nationwide Inpatient Sample (NIS) database [9]. Both of these studies relied on International Classification of Diseases (ICD) diagnosis codes for AH in an administrative database for case identification. Although such codes have been validated for other non-hepatic and hepatic conditions (e.g. cirrhosis, viral hepatitis, autoimmune liver disease, drug hepatotoxicity) [10–21], their validity for AH has yet to be confirmed. While the burden of alcohol-related liver disease is increasing in many regions, large-scale studies describing the epidemiology of AH in Canada have been hindered by the lack of a validated coding algorithm. Therefore, the primary objective of our study was to validate coding algorithms for the identification of patients hospitalized for AH using administrative data. Our study findings will inform researchers if administrative data can be used for epidemiological studies and surveillance of this condition.

## Methods

### Study population

In this retrospective study, the hospital Discharge Abstract Database (DAD) was queried to identify adults (age ≥18 years) hospitalized in the Calgary region in the province of Alberta, Canada with a primary or secondary diagnosis of AH (ICD-10 diagnosis code, K70.1 [22]) between January 2008 and August 2012. Only the first hospitalization was considered among patients with multiple admissions for AH to avoid selection bias. Specifically, patients diagnosed with AH are more likely to be diagnosed with AH again during future admissions, thereby falsely elevating coding validity. The Calgary region included three adult, acute care hospitals and served a catchment population of approximately 1.5 million individuals. Over 99 % of Alberta residents are registrants of the Alberta Health Care Insurance Plan, a universal plan that covers all hospitalization costs [23]. The DAD contains up to 25 diagnoses, 20 procedures, and mortality information on all discharges from Calgary hospitals. This database has been used to examine the epidemiology [24, 25], outcomes [26–33], and coding accuracy [18, 24] of various medical conditions. This study protocol and a waiver of consent was approved by the Conjoint Health Research Ethics Board at the University of Calgary.

### Data extraction and definition of AH

Admissions were randomly assigned to one of three physicians experienced in the care of patients with AH (MB, ER, JP) who reviewed the paper and electronic medical records for each individual to confirm the diagnosis of AH during their index admission. Using a structured data collection instrument, the following details were recorded: age, sex, year and site of hospitalization, liver biochemistry (serum alanine aminotransferase [ALT], aspartate aminotransferase [AST], gamma-glutamyl-transpeptidase [GGT], bilirubin, albumin concentrations, and international normalized ratio [INR]) and serum creatinine at admission, clinical evidence of hepatic encephalopathy (based on the impression of the attending physician) or ascites (detected clinically or radiologically), and self-reported recent average daily alcohol intake. Since alcohol intake varied throughout some medical records, a hierarchical approach was taken. Specifically, data was first taken from consultation notes by addictions specialists where available, followed by the admission history, and otherwise, from patient progress notes. The severity of hepatic dysfunction was described using the Model for End-Stage Liver Disease (MELD) score [34] and Maddrey DF, validated prognostic scoring systems for patients with AH. Patients with a Maddrey DF ≥32 and/or the presence of hepatic encephalopathy were classified as severe [6]. Length of stay (LOS) and mortality (in-hospital and at 90 and 180 days) were also recorded.

Although the reference standard for the diagnosis of AH includes liver biopsy, this procedure is not part of the routine clinical management of patients with suspected AH in Calgary hospitals. Therefore, patients were considered to have a confirmed diagnosis of AH if they fulfilled all the following criteria based on history and laboratory investigations at hospital admission: 1) heavy alcohol consumption (>196 g/week or >56 g in any day among males, and >98 g/week or >42 g in any day among females) [35]; 2) elevated serum AST and/or ALT concentration, but <300 IU/L (to exclude other disorders associated with acute hepatic dysfunction including acetaminophen toxicity); 3) serum bilirubin >34 μmol/L; 4) elevated INR; and 5) exclusion of other causes of acute hepatic dysfunction (e.g. drug hepatotoxicity, autoimmune hepatitis, ischemic hepatitis, etc.). Patient with missing information on any of the above criteria are considered “Not AH”. To confirm the inter-rater agreement of a diagnosis of AH according to these criteria, 21 patients were randomly selected and all three physicians reviewed their medical records. In cases of disagreement, a consensus was reached.

### Statistical analyses

Between groups comparisons were made using Fisher's exact and χ^2^ tests for categorical variables, and Wilcoxon rank-sum test for continuous variables. Using data from medical records as the reference standard for the diagnosis of AH, we calculated the positive predictive value (PPV) for each administrative data coding algorithm with exact binomial 95 % confidence intervals (CI). Due to the lack of an unaffected control group, we could not calculate the sensitivity, specificity, or negative predictive values of these algorithms. Subgroup analyses of algorithm accuracy were performed according to the diagnosis field in which the code for AH was recorded (primary vs. secondary) and AH severity (mild vs. severe), with a severe presentation defined by a Maddrey DF of ≥32 and/or the presence of hepatic encephalopathy. Further stratification by fiscal year and admitting hospital was performed to assess for any temporal changes in coding accuracy or heterogeneity between hospitals, which employ different health records coders. In addition, the diagnostic accuracy of algorithms that incorporated ICD-10 diagnosis codes for cirrhosis and its complications (i.e. ascites, gastrointestinal hemorrhage, hepatic encephalopathy, malnutrition, hepatorenal syndrome/renal failure) and alcohol-related disorders (i.e. alcohol abuse, alcohol dependence, alcohol withdrawal, and pancreatitis) were examined (see Appendix for codes). Algorithms that accounted for the number of associated conditions were also assessed. Finally, inter-rater agreement for the diagnosis of AH, with subgroup analyses according to disease severity, was calculated using the kappa statistic [36]. All analyses were performed using Stata version 11.0 (StataCorp; College Station, TX, USA). Two-sided *P*-values less than 0.05 were considered statistically significant.

## Results

### Patient characteristics

A total of 228 patients were hospitalized in the Calgary region between January 2008 and August 2012 with a diagnosis code for AH (71 % [*n* = 161] as the primary diagnosis). Their characteristics are outlined in Table 1. The median age was 49 years (interquartile range [IQR] 43–55) and 61 % of the cohort was male. A similar proportion was hospitalized at each of the three adult hospitals in Calgary (*P* = 0.69). The majority of cases (62 %, *n* = 142) had a severe presentation. Overall, median length of stay was 7 days (IQR 5–15) and in-hospital mortality was 6 % (*n* = 13). Compared with those with a mild presentation, patients with a severe presentation had a prolonged median LOS (5 vs. 10 days; *P* < 0.001) and greater in-hospital mortality (0 % vs. 9 %; *P* = 0.002).

**Table 1:** Characteristics of the study population

	Entire cohort (*n* = 228)	Confirmed AH cases (*n* = 122)	Unconfirmed AH cases (*n* = 106)	*P*-value
Age, *years*	49 (43–55)	49 (42–55)	49 (44–58)	0.33
Male	61 % (138)	60 % (73)	61 % (65)	0.89
Self-reported alcohol consumption, *g/day*	119 (81–224)	121 (84–233)	112 (56–208)	0.024
Excessive intake ^a^	91 % (208)	100 % (122)	81 % (86)	<0.001
Proportion in the highest intake quartile	25 % (50)	25 % (29)	24 % (21)	0.87
Admission hospital				0.69
1	39 % (90)	42 % (51)	37 % (39)	
2	30 % (68)	29 % (36)	31 % (32)	
3	31 % (70)	29 % (35)	33 % (35)	
Year of admission				0.16
2008	25 % (56)	22 % (27)	27 % (29)	
2009	17 % (40)	15 % (18)	21 % (22)	
2010	18 % (41)	17 % (21)	19 % (20)	
2011	21 % (48)	21 % (26)	21 % (22)	
2012	19 % (43)	25 % (30)	12 % (13)	
Biochemical status				
Maddrey DF	29 (13–52)	45 (26–62)	12 (5–30)	<0.001
MELD	18 (13–23)	21 (18–24)	13 (8–19)	<0.001
Severe ^b^	62 % (142)	80 % (97)	42 % (45)	<0.001
Associated conditions				
Cirrhosis	25 % (58)	29 % (35)	22 % (23)	0.29
Ascites	27 % (61)	38 % (46)	14 % (15)	<0.001
GI hemorrhage	34 % (77)	39 % (48)	27 % (29)	0.07
Hepatic encephalopathy	2.6 % (6)	2.5 % (3)	2.8 % (3)	1
Malnutrition	3.0 % (7)	4.1 % (5)	1.9 % (2)	0.45
HRS/renal failure	16 % (36)	18 % (22)	13 %(14)	0.37
Pancreatitis	7.9 % (18)	4.9 % (6)	11 % (12)	0.09
Alcohol abuse	36 % (81)	38 % (46)	33 % (35)	0.49
Alcohol dependence	26 % (60)	28 % (34)	25 % (26)	0.65
Alcohol withdrawal	21 % (47)	16 % (19)	26 % (28)	0.05
Mortality				
In-hospital	5.7 % (13)	7.4 % (9)	3.7 % (4)	0.27
90 days	13 % (29)	17 % (21)	8.5 % (9)	0.045
180 day	17 % (38)	23 % (28)	11 % (12)	0.007

Data are presented as median (IQR) or % (n). *DF* discriminant function, *GI* gastrointestinal, *HRS* hepatorenal syndrome; MELD, Model for End-Stage Liver Disease

^a^ As defined in the Methods

^b^ Severe presentation is characterized by a Maddrey DF of ≥32 and/or the presence of hepatic encephalopathy

### Validation of an AH diagnosis based on administrative data

Of the 228 potential AH cases, 122 patients had confirmed AH based on medical record review, corresponding to a PPV of 54 % (95 % CI 47-60 %). Overall, inter-rater agreement for an AH diagnosis was good (kappa 0.86; *P* < 0.001), and higher among patients with a severe (kappa 0.88; *P* < 0.001) versus mild (kappa 0.80; *P* < 0.002) presentation. Compared to unconfirmed cases, patients with confirmed AH had greater liver disease severity according to the Maddrey DF (45 vs. 12; *P* < 0.001) and MELD scores (21 vs. 13; *P* < 0.001); a higher prevalence of ascites (38 % vs. 14 %; *P* < 0.001); a lower prevalence of alcohol withdrawal (16 % vs. 26 %; *P* = 0.05); and greater 90-day and 180-day mortality (Table 1). Patients with confirmed AH also had greater median daily alcohol intake (121 vs. 112 g; *P* = 0.024), however, there was no significant difference in the proportion of patients that had daily alcohol intake within the top quartile (24 % vs. 25 %; *P* = 0.87). Differences between confirmed and unconfirmed AH cases were not observed with respect to age, sex, admitting hospital, year of admission, or the remaining cirrhosis and alcohol-related complications (Table 1).

### Subgroup analyses of AH coding algorithm validity

Among confirmed AH cases, 89 % (108/122) had an AH code recorded as the primary diagnosis compared with only 50 % (53/106) among unconfirmed cases (*P* < 0.001). The PPV improved from 54 % overall to 67 % (95 % CI 59-74 %) when AH was the primary diagnosis versus 21 % (95 % CI 12-33 %) when AH was recorded as a secondary diagnosis (*P* < 0.001). A severe presentation was also more common in patients with confirmed AH versus unconfirmed cases (80 % [97/122] vs. 43 % [45/106]; *P* < 0.001). Accordingly, the PPV of an AH diagnosis was higher among severe compared to mild cases (68 % [95 % CI 60-76 %] vs. 29 % [95 % CI 20-40 %]; *P* < 0.001). When stratified by year of hospitalization, the PPV was highest in 2012 (70 %; 95 % CI 54-83 %) compared to 2008 through 2011 (45 % to 54 %), however, this difference was not statistically significant (*P* = 0.17). Comparison of the PPV between admitting hospitals, which ranged from 50 % to 57 %, showed no significant differences between centres (*P* = 0.69).

The PPVs of diagnostic algorithms that included codes for cirrhosis and alcohol-related conditions in the administrative data are shown in Table 2. The PPVs tended to be higher for algorithms that included ascites (75 %; 95 % CI 63-86 %), gastrointestinal hemorrhage (62 %; 95 % CI 51-73 %), and cirrhosis (60 %; 95 % CI 47-73 %). In general, these PPVs improved when restricted to cases with an AH code in the primary diagnosis field and those with severe presentations (Table 2). However, the prevalence of these conditions in patients with confirmed AH was low, ranging from 29 % for cirrhosis to 39 % for gastrointestinal hemorrhage. The prevalence of codes for other cirrhosis and alcohol-related disorders were generally too low or their inclusion did not enhance the PPV of the algorithms (Table 2). For example, a diagnosis code for hepatic encephalopathy was recorded in only 2.5 % of confirmed AH cases (4.2 % of severe cases), and thus, the PPV of an algorithm including both an AH and hepatic encephalopathy code was only 50 % (95 % CI 12-88 %). Stratification by year and hospital of admission did not indicate any consistent differences between PPVs for these algorithms (data not shown).

**Table 2:** Prevalence and performance characteristics of algorithms including an alcoholic hepatitis code and codes for associated conditions

	Prevalence % (*n*)		Positive predictive value (PPV) % (95 % CI)				
Condition	Overall (*n* = 22	Confirmed AH (*n* = 122)	Overall (*n* = 228	AH as Primary Diagnosis (*n* = 161)	AH as Secondary Diagnosis (*n* = 67)	Severe AH (*n* = 142)	Mild AH (*n* = 86)
Ascites	27 % (61)	38 % (46)	75 % (63–86)	78 % (65–89)	6 % (26–88)	83 % (70–93)	46 % (19–75)
GI hemmorrhage	34 % (77)	39 % (48)	62 % (51–73)	76 % (62–87)	30 % (13–53)	80 % (66–90)	30 % (14–50)
Hepatic encephalopathy	2.6 % (6)	2.5 % (3)	50 % (12–88)	100 % (16–100)	25 % (1–81)	50 % (12–88)	---
Cirrhosis	25 % (58)	29 % (35)	60 % (47–73)	70 % (53–83)	39 % (17–64)	69 % (53–82)	31 % (9–61)
Alcoholic hepatic failure	4.8 % (11)	8.2 % (10)	91 % (59–100)	89 % (52–100)	44 % (20–70)	90 % (55–100)	33 % (10–65)
Malnutrition	3.0 % (7)	4.1 % (5)	71 % (29–96)	60 % (15–95)	100 % (16–100)	80 % (28–99)	50 % (1–99)
HRS/ Renal failure	16 % (36)	18 % (22)	61 % (43–77)	67 % (46–83)	44 % (14–79)	68 % (48–84)	38 % (9–76)
Pancreatitis	7.9 % (18)	4.9 % (6)	33 % (13–59)	63 % (24–91)	10 % (0–45)	86 % (42–100)	0 % (0–28)
Alcohol abuse	35 % (81)	38 % (46)	57 % (43–68)	68 % (54–79)	27 % (11–50)	70 % (56–82)	32 % (16–52)
Alcohol dependence	26 % (60)	28 % (34)	57 % (43–69)	74 % (60–86)	17 % (4–41)	76 % (59–88)	26 % (10–48)
Alcohol withdrawal	21 % (47)	16 % (19)	40 % (26–56)	62 % (41–80)	14 % (3–36)	62 % (41–80)	14 % (3–36)

*GI* gastrointestinal, *HRS* hepatorenal syndrome

Table 3 outlines the performance characteristics of algorithms according to the number of cirrhosis and alcohol-related conditions recorded in the administrative data in addition to an AH diagnosis code. In patients with three or more associated conditions, the PPV for confirmed AH improved to 62 % to 79 %; however, only 6 % to 34 % of confirmed cases had this number of additional diagnoses in their administrative data.

**Table 3:** Performance characteristics for algorithms for alcoholic hepatitis according to the number of associated conditions ^a^

Number of conditions ^a^	Prevalence % (*n*)		Positive predictive value (PPV) % (95 % CI)
	Overall (*n* = 228)	Confirmed AH (*n* = 122)	
0	11 % (24)	7.4 % (9)	38 % (19–59)
≥1	89 % (204)	93 % (113)	55 % (48–62)
≥2	61 % (139)	65 % (79)	57 % (48–65)
≥3	30 % (68)	34 % (42)	62 % (49–73)
≥4	13 % (29)	19 % (23)	79 % (60–92)
≥5	4 % (9)	5.7 % (7)	78 % (40–97)

^a^ Associated conditions as shown in Table 2. Prevalent cases represent those with an AH diagnosis code plus codes for the associated conditions

## Discussion

In this population-based study, we assessed the validity of ICD diagnosis coding algorithms for AH in a Canadian hospitalization database. Our finding of a low PPV for AH (54 %) suggests that AH was not accurately and completely coded in the administrative data and caution must be exercised if the DAD is used for large-scale epidemiologic studies of AH. Under the assumption that the PPV remains relatively constant over time (as observed in our study), the DAD could be used to assess temporal trends in AH admissions. However, the exact incidence for any given year based on this data is likely to be erroneous. Administrative data could also be used as a screening tool for potential cases with AH. In this situation, confirmation of the presence of AH requires additional clinical information such as that obtainable via a review of medical records. In light of our findings, the validity of previous studies that used administrative data to study the epidemiology and outcomes of AH should be scrutinized. In one study, Sandahl and colleagues used this ICD-10 code to identify AH cases from the Danish National Registry of Patients and describe its incidence and associated mortality [8]. In total, 1,951 suspected cases of AH were identified between 1999 and 2008. The annual incidence increased from 37 to 46 per million population in men and from 24 to 34 per million in women. However, according to our results, close to half of the patients identified in this study may not have been true cases of AH. This study also reported 84-day mortality ranging from 14 % to 24 %, similar to the 90-day mortality rate of 17 % for confirmed AH in our study. In another study, Liangpunsakul used the Nationwide Inpatient Sample hospitalization database to report the outcomes of patients hospitalized for AH in the United States with case identification using the ICD-9 code for this condition (571.1) [9]. The study showed that AH represented 0.71 % of all hospital admissions in 2007 and reported an in-hospital mortality rate of 6.8 %. Although our study validated only the ICD-10 coding classification (as ICD-9 codes were unavailable), there is no clear rationale for the accuracy to differ among the two classification systems both of which include a single code for AH. Moreover, a study by Quan and colleagues that used the DAD (as in our study) demonstrated that the coding accuracies between the ICD-9 and ICD-10 classifications for liver diseases are very similar (AH was not examined specifically in this study) [37]. Therefore, our results should be generalizable to databases using either the ICD-9 or ICD-10 classifications.

The diagnosis field in which the code for AH was recorded in the administrative database (i.e. primary vs. secondary) had a significant impact on algorithm validity. Indeed, the majority (89 %) of confirmed AH cases had the AH code recorded as the primary diagnosis (vs. 50 % among non-AH hospitalizations). Accordingly, the PPV improved from 54 % to 67 % when the cohort was restricted to those with AH as the primary diagnosis compared with one of the secondary diagnosis fields. Patients with a severe presentation were also more likely to have AH recorded as the primary diagnosis. Specifically, 77 % of cases with hepatic encephalopathy and/or a Maddrey DF ≥32 had AH as a primary diagnosis compared with only 59 % of those with mild hepatic dysfunction. Algorithms that also included diagnosis codes for ascites or gastrointestinal hemorrhage had improved performance, particularly when restricted to cases with a primary diagnosis of AH in which PPVs of 76 % to 78 % were observed. The corollary is that studies focused on patients with severe AH may potentially identify these cases from administrative databases by restricting to cases with AH as the primary diagnosis plus these associated conditions. However, the prevalence of these cirrhosis-related complications was low enough (<40 %) in confirmed AH cases that any study employing this methodology will have reduced sensitivity for the identification of all relevant cases. Based on the sub-optimal accuracy observed in our study, we would advise that any AH case identified in this manner be confirmed via a review of medical records.

In addition to examining the impacts of coding details and disease severity on the validity of an AH code in the administrative data, we studied the effects of year and hospital of admission. Despite the presence of different health records coders at the three hospitals in our region, coding validity was similar supporting the generalizability of our findings. Moreover, the PPV of an AH code did not differ across study years, potentially supporting the temporal trends in disease incidence suggested by the study of Sandahl and colleagues [8]. In this regard, we did not observe an increase in the number of admissions over time although the sample size of our study was limited.

While previous studies have confirmed the validity of administrative data for the identification of patients with various liver disorders [19, 38, 39], our results suggest that caution is needed when using these data sources to study AH. However, the major challenge with our analysis relates to the diagnostic definition for AH. Specifically, AH represents a spectrum ranging from mild abnormalities in liver biochemistry to life-threatening liver failure due to abusive alcohol consumption when other causes of liver disease (e.g. viral hepatitis and drug hepatotoxicity) have been excluded. Although AH has characteristic histological findings including steatosis, ballooned hepatocytes, Mallory bodies, lobular neutrophilc inflammation, and centrizonal fibrosis [40], liver biopsy is not routinely performed in our region, nor are these findings specific (e.g. they may be seen in patients with non-alcoholic steatohepatitis). The diagnostic criteria suggested by Lucey et al. (i.e. elevated AST but <300 IU/L, AST to ALT ratio >2, serum bilirubin >86 μmol/L, elevated INR, and neutrophilia in patients with ascites and a history of heavy alcohol use) [1] are very stringent and would mostly capture only patients with severe AH. In light of this fact, we utilized a less rigorous definition of AH that allowed us to identify a wider spectrum of suspected AH cases. Moreover, since AST is not part of standard liver biochemical profiles in our region and due to the high correlation between AST and ALT, we considered ALT in place of AST when necessary. Nevertheless, since such a biochemically focused, even more lenient, case definition is generally not strictly followed in clinical practice, patients who do not meet all of these criteria may still be diagnosed with AH by their physician thereby contributing to the sub-optimal accuracy of the diagnosis code observed in our study.

Among the 106 patients who did not fulfill our diagnostic criteria for confirmed AH, 31 patients clearly did not have AH upon medical records review. The diagnosis for these patients includes cirrhosis, sepsis, recent history of AH, pancreatitis, acetaminophen toxicity, cocaine use, heart failure and alcohol intoxication.

Taking into consideration that our definition of AH may not be strictly followed by physicians, we performed a sensitivity analysis using a loosened definition of AH that confirmed diagnosis of AH if patients fulfilled both of the following criteria: heavy alcohol consumption (>196 g/week or >56 g in any day among males, and >98 g/week or >42 g in any day among females) and exclusion of other causes of acute hepatic dysfunction (e.g. drug hepatotoxicity, autoimmune hepatitis, ischemic hepatitis, etc.). With this loosened diagnostic criteria, PPV improved to 73 % (95 % CI 67-79 %), up from 54 %. This improved PPV remains sub-optimal, thus further supporting our initial findings that AH was not accurately and completely coded in the administrative data. Realizing the limitations of both sets of diagnostic criteria, one could infer that the true PPV for the AH diagnosis code lies within 54 % to 73 %.

Our study has several limitations that warrant discussion. First, we lack data for a control group representative of the general hospitalized population. Without controls, the sensitivity, specificity and NPVs of the diagnosis code for AH (and related algorithms) cannot be calculated. Second, since this is a retrospective study, the reliability of patients’ self-reported alcohol intake is questionable. On numerous occasions, multiple descriptions of alcohol consumption were recorded in the medical record. Anecdotally, patients tended to admit to greater alcohol intake when questioned by addictions specialists. As a result, we utilized a hierarchical approach to record alcohol consumption (see Methods). Since the amount of alcohol intake is a vital criterion in the reference standard for an AH diagnosis, underreporting may have led to an underestimation of AH in our study.

## Conclusions

In conclusion, the validity of ICD-10 coding for AH in this Canadian hospitalization database appears sub-optimal. Although the accuracy of coding algorithms improved when restricted to patients with AH in the primary diagnosis field or with codes indicative of hepatic complications (e.g. ascites and gastrointestinal haemorrhage), the use of administrative data in epidemiologic studies of AH should be undertaken cautiously. If administrative data is used for case identification, confirmation of the diagnosis via medical record review is recommended.

### Availability of data and materials

Not applicable.

## Appendix

**Table 4:** Codes used to identify associated conditions

Condition	ICD-10 diagnosis codes	CCI procedure codes [41]
Alcoholic hepatitis	K70.1	
Ascites	R18	1OT52HA, 1OT52HAT, 1OT52LAT
GI hemorrhage	K92.0, K92.1, K92.2, K62.5, I85.0, I85.9, I98.20, I98.21, I98.3, I86.4, K29.0, K29.2, K29.5, K29.7, K29.8. K20, K21.0, K25.0, K25.3, K25.4, K25.7, K25.9, K26.4, K26.9, K27.9, K22.6, K22.1	1NA13BAH, 1NA13BAX, 1NA13BAF, 1NF13BAK
Hepatic encephalopathy	K72	
Cirrhosis	K70.3, K74.6	
Alcoholic hepatic failure	K70.4	
Malnutrition	E41, E43, E46	
HRS/renal failure	K76.7, N17.0, N17.8, N17.9	1PZ21HPD, 1PZ21HQB
Pancreatitis	K85, K85.2, K85.9, K86.0, K86.1	
Alcohol abuse	F10.1	
Alcohol dependence	F10.2	
Alcohol withdrawal	F10.3	

*GI* gastrointestinal, *HRS* hepatorenal syndrome, *CCI* Canadian Classification of Health Interventions

## Abbreviations

AH: Alcoholic hepatitis
ALT: Alanine aminotransferase
AST: Aspartate aminotransferase
CI: Confidence interval
CIHI: Canadian Institute of Health Information
GI: Gastrointestinal
HE: Hepatic encephalopathy
ICD: International statistical classification of disease
INR: International normalized ratio
IQR: Interquartile range
NIS: Nationwide Inpatient Sample
PPV: Positive predictive value
